# Enthusiasm Scientifically Oriented: The Preface for the Special Issue Dedicated to Jan Albrecht

**DOI:** 10.1007/s11064-016-2164-x

**Published:** 2017-01-11

**Authors:** Magdalena Zielińska, Michael Aschner

**Affiliations:** 1 Department of Neurotoxicology, Mossakowski Medical Research Centre, Polish Academy of Sciences, 5 A. Pawińskiego Street, 02-106 Warsaw, Poland; 2 Department of Molecular Pharmacology, Albert Einstein College of Medicine, Forchheimer 209; 1300 Morris Park Avenue, Bronx, NY 10461 USA


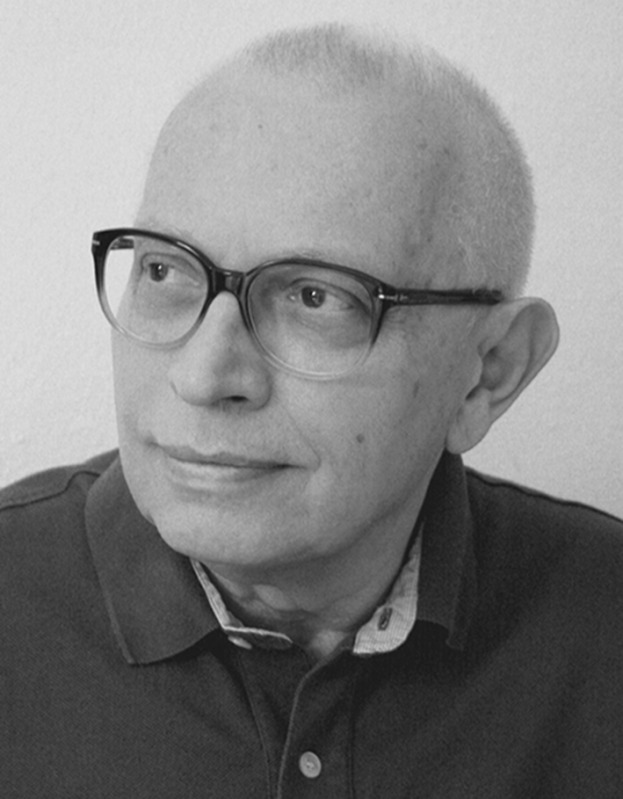


It is a privilege to write this preface in the special issue of *Neurochemical Research* dedicated to Professor Jan Albrecht, a truly enthusiastic biochemist forever bursting with ideas, always giving the impression that he is permanently dealing with a *plethora* of different topics and pursuing countless amounts of activities simultaneously. At seventy-two, he simply continues to be the person he has been all through his life: an outspoken and often uncompromising researcher with an incredibly sharp analytical skill, an internationally acknowledged expert in many different fields of neurochemistry.

Professor Albrecht was born in 1944 in Warsaw and it was there he received his education. He is a graduate of biochemistry from the Department of Biology, University of Warsaw, and after being a voluntary assistant at the State University of Leiden (The Netherlands), he obtained his PhD in biochemistry there at the Department of Chemistry. Back in Poland, he joined the Department of Neuropathology in the Mossakowski Medical Research Centre (MMRC), Polish Academy of Sciences in Warsaw. In 1980 he obtained “doctor habilitatus” (second doctorate) in molecular biology (Institute of Immunology and Experimental Therapy, Wrocław, Poland). In 1996, he became the head of the Department of Neurotoxicology in the Mossakowski Medical Research Centre, PAS, serving there since 2014 as Professor Emeritus.

In the early 1980s, Professor Albrecht was one of the first to recognize the role of astrocytic dysfunction in the pathogenesis of hyperammonemia and hepatic encephalopathy. Before that, in the Leiden period, Professor Albrecht characterized in detail the ribosomal initiation factor causing dissociation of bacterial ribosomes to the 30 S and 50 S subunit [1]. The pioneering observations made by Professor Albrecht are related to the role of astrocytes in the healthy and diseased brain. Professor Albrecht demonstrated the presence of glutamate and GABA receptors in these cells, [2, 3] and of active transport of glutamine in exchange of glutamate [4]. Those days his scientific interests also focused on the mechanisms underlying ammonia neurotoxicity and gliotoxicity in hepatic encephalopathy. Working independently, Professor Albrecht and Professor Michael D. Norenberg demonstrated that glutamine accumulating in excess in the cells under hyperammonemia causes mitochondrial damage and astrocytic swelling which leads to brain edema. Based on these discoveries, Professor Albrecht and Professor Norenberg formulated the “Trojan horse” hypothesis emphasizing the “Dr Jekyll and Mr Hyde” role of this amino acid [5]. His subsequent continuous research interests include interrelated roles of glutamine and oxidative/nitrosative stress in hepatic encephalopathy.

The scientific work of Professor Albrecht has led to more than 220 publications and chapters, which have been cited more than 3500 times (current H-index 30). His research has brought him international recognition. Professor Albrecht is an outstanding lecturer, who has delivered dozens of lectures around the world at prestigious meetings, including the very last Hans Prydz Lecture in University of Oslo in the year 2012. Professor Albrecht served and continues to serve as the valued member of editorial boards in many top-ranking journals, including *Neurochemical Research, Journal of Neuroscience Research, Neurochemistry International, Frontiers in Bioenergetics, Metabolic Brain Disease, Molecular and Chemical Neuropathology and Journal of Neurochemistry*. He is a member of learned societies and committees such as the Polish Academy of Sciences and Art, the Polish Academy of Sciences, the American Society for Neurochemistry, the European Society for Neurochemistry, the International Society for Developmental Neurosciences, the Polish Neuroscience Society, the Committee of Neurobiology PAS, the Committee of Physiological Sciences PAS. Since 2013, Professor Albrecht is a member of Academia Europaea. He also serves as a member on many research councils, which is highly esteemed by the scientific community. Among the prizes that he has received are the Award of the Prime Minister of Poland for outstanding achievements in science and the Award of the Foundation for Polish Science (Master Programme).

Wide scientific international contacts allowed Professor Albrecht to co-organize and co-chair “Wierzba Conferences”, a cyclic triennial event gathering top European and overseas experts in the field of glutamate metabolism and function in healthy and diseased brain (Wierzba, Poland; 1999; 2002; 2005; 2008; Aghia Pelagia, Greece, 2011; Cracow, 2014). One looks forward with great anticipation to the next scientific contributions that Professor Albrecht will make in the coming years.

To honor Professor Albrecht, this Special Issue comprises contributors that reflect on the broad range of his interests in neurochemical research. He will be particularly thrilled to note that so many of the researchers in the field who are his long-term friends and collaborators participated in the *creation* of this issue published in his honor.

Magdalena Zielińska & Michael Aschner

Guest Editors
